# Medium-Chain Triglycerides Attenuate Liver Injury in Lipopolysaccharide-Challenged Pigs by Inhibiting Necroptotic and Inflammatory Signaling Pathways

**DOI:** 10.3390/ijms19113697

**Published:** 2018-11-21

**Authors:** Lin Zhang, Xiuying Wang, Shaokui Chen, Shuhui Wang, Zhixiao Tu, Guolong Zhang, Huiling Zhu, Xiangen Li, Jianglin Xiong, Yulan Liu

**Affiliations:** 1 Hubei Collaborative Innovation Center for Animal Nutrition and Feed Safety, Hubei Key Laboratory of Animal Nutrition and Feed Science, Wuhan Polytechnic University, Wuhan 430023, China; lynnemumu@126.com (L.Z.); xiuyingdk@126.com (X.W.); yb77526@umac.mo (S.C.); 15827239218@163.com (S.W.); 18771099654@163.com (Z.T.); glenn.zhang@okstate.edu (G.Z.); zhlsy7114@163.com (H.Z.); genuncle@163.com (X.L.); xiongjianglin@126.com (J.X.); 2 Department of Animal Science, Oklahoma State University, Stillwater, OK 74078, USA

**Keywords:** medium-chain triglycerides, LPS, liver injury, necroptosis, inflammation

## Abstract

This study was conducted to investigate whether medium-chain triglycerides (MCTs) attenuated lipopolysaccharide (LPS)-induced liver injury by down-regulating necroptotic and inflammatory signaling pathways. A total of 24 pigs were randomly allotted to four treatments in a 2 × 2 factorial design including diet (0 and 4% MCTs) and immunological challenge (saline and LPS). After three weeks of feeding with or without 4% MCTs, pigs were challenged with saline or LPS. MCTs led to a significant increase in eicosapentaenoic acid, docosahexaenoic acid and total (n-3) polyunsaturated fatty acid concentrations. MCTs attenuated LPS-induced liver injury as indicated by an improvement in liver histomorphology and ultrastructural morphology of hepatocytes, a reduction in serum alanine aminotransferase and alkaline phosphatase activities as well as an increase in claudin-1 protein expression. In addition, MCTs also reduced serum tumor necrosis factor-α (TNF-α), interleukin (IL)-1β and IL-6 concentrations, liver TNF-α and IL-1β mRNA expression and protein concentrations and enhanced liver heat shock protein 70 protein expression in LPS-challenged pigs. Moreover, MCTs decreased mRNA expression of receptor-interacting serine/threonine-protein kinase (*RIP*) 3, mixed-lineage kinase domain-like protein (*MLKL*) and phosphoglycerate mutase 5 and inhibited MLKL phosphorylation in the liver. Finally, MCTs decreased liver mRNA expression of toll-like receptor (*TLR*) 4, nucleotide-binding oligomerization domain protein (*NOD*) 1 and multiple downstream signaling molecules. MCTs also suppressed LPS-induced p38 mitogen-activated protein kinase (MAPK) phosphorylation and increased extracellular signal-related kinase 1/2 phosphorylation in the liver. These results indicated that MCTs are capable of attenuating LPS-induced liver damage by suppressing hepatic necroptotic (RIP1/RIP3/MLKL) and inflammatory (TLR4/NOD1/p38 MAPK) signaling pathways.

## 1. Introduction

The liver is a vital organ with a wide range of metabolic, detoxification and endocrine functions. The liver is also an important immune organ with a unique population of cells that participate in innate and adaptive immune responses [1]. Various bacterial and viral infections, such as acute hepatitis, as well as toxins can result in parenchymal liver injury and dysfunction [2]. Lipopolysaccharide (LPS), a potent endotoxin, plays a critical role in many liver diseases such as alcoholic steatohepatitis [3], nonalcoholic steatohepatitis [4], non-alcoholic fatty liver disease (NAFLD) [4], liver cirrhosis [5] and ischemic liver injury [6]. LPS stimulates liver macrophages (Kupffer cells) to produce various pro-inflammatory cytokines, which leads to inflammatory response and liver injury [7]. Nutritional interventions have been investigated for their hepatoprotective effects.

Medium-chain fatty acids with 6–12 carbon atom chains (e.g., caproic acid, octanoic acid, capric acid and lauric acid) occur naturally as medium-chain triglycerides (MCTs). MCTs are abundant in milk fat and several plant oils including coconut oil, palm oil and *Cuphea* seed oil. MCTs are hydrolyzed by gastric and pancreatic lipases in neonates and nursing animals and provide instant energy for enterocytes and liver metabolism [8]. Due to their ability to be absorbed rapidly by the body, MCTs are important in patients with absorption disturbances such as diarrhea, steatorrhea, celiac disease and digestion problems due to partial surgical removal of the stomach or the intestine [9]. MCTs are also used in nutrition of prematurely born infants due to their immature digestive tract and high energy demand [10]. In addition, MCTs are used for athletes’ nutritional support during training, to increase exercise performance, decrease body fat and increase lean muscle mass [11]. Moreover, there is evidence that MCTs are hepatoprotective in animal models of liver injury and clinical trials. Li et al. (2013) reported that feeding with MCT prevented alcohol-induced hepatic lipid dyshomeostasis in rats [12]. Ronis et al. (2013) found that MCT prevented liver pathology in a rat model of NAFLD [13]. MCT has also been shown to prevent alcohol-induced liver injury in clinically in patients with cirrhosis [14].

Necroptosis, a novel form of caspase-independent cell death, has been implicated in the development of several liver diseases [15,16,17]. The morphology of cells undergoing necroptosis is similar to that of necrotic cells but differs from cells undergoing apoptosis. However, unlike necrosis, necroptosis is a highly regulated process involving receptor-interacting serine/threonine-protein kinase (RIP)1, RIP3 and mixed-lineage kinase domain-like protein (MLKL) [18,19]. If cellular caspases are inhibited, RIP1 interacts with RIP3 to form necrosomes, amyloid-like structures that are stabilized by phosphorylation of both kinases [19]. Activated RIP3 then recruits and phosphorylates MLKL to promote its oligomerization and translocation to the plasma membrane, resulting in membrane rupture, necrosis and tissue damage [18].

Necroptosis is a highly proinflammatory mode of cell death that leads to rapid generation of damage-associated molecular patterns (DAMPs) comprised of proteins that promote noninfectious inflammation [18]. Toll-like receptors (TLRs) and nucleotide-binding oligomerization domain proteins (NODs) are two families of pattern recognition receptors, which are involved in inflammation by recognizing DAMPs or pathogen-associated molecular patterns (PAMPs) [20]. Activation of both TLRs and NODs by specific PAMPs or DAMPs results in the subsequent activation of nuclear factor-κB (NF-κB) and mitogen-activated protein kinases (MAPKs) that trigger increased production of proinflammatory cytokines such as tumor necrosis factor-α (TNF-α), interleukin (IL)-1β and IL-6 and lead to tissue injury [20].

Because both necroptosis and inflammation are major players in liver injury under pathological conditions, we hypothesized that dietary MCTs would alleviate liver damage by suppressing necroptosis and inflammation through down-regulation of RIP1/PIP3/MLKL-, TLR- and NOD-mediated signaling pathways. In this study, a well-established pig model of acute LPS-induced hepatic injury [7,21,22] was used to investigate the molecular mechanisms by which MCTs exerted a hepatoprotective effect.

## 2. Results

### 2.1. MCT Supplementation Has No Effect on Growth Performance before LPS Challenge

During the whole experiment period, there were no adverse events. In the 21 days of feeding prior to LPS challenge or saline injection, no differences were observed in the initial (9.1 ± 0.2 kg) and final (20.6 ± 0.5 kg) body weight (BW), average daily gain (498 ± 26 g), average daily feed intake (745 ± 30 g), or feed/gain ratio (1.50 ± 0.04) in the control and MCT-supplemented groups.

### 2.2. MCT Supplementation Affects Liver Fatty Acid Composition after LPS Challenge

LPS challenge had no significant effect on liver fatty acid composition (Table S1). No LPS challenge × diet interaction was found for the levels of palmitoleic acid (C16:1), linoleic acid (C18:2n-6), or total (n-6) polyunsaturated fatty acids (PUFAs) in the liver. However, pigs fed with MCTs had higher level of palmitoleic acid and lower levels of linoleic acid and total (n-6) PUFAs compared with the pigs fed the control diet (*P* ≤ 0.001). An LPS challenge × diet interaction was observed for eicosapentaenoic acid (EPA, C20:5n-3), docosahexaenoic acid (DHA, C22:6n-3), total (n-3) PUFAs and the (n-6)/(n-3) ratio (*P* < 0.01). A trend for an LPS challenge × diet interaction was observed for oleic acid (C18:1n-9) (*P* = 0.06). Relative to the control diet, MCTs increased the oleic acid content in LPS-challenged pigs, DHA and total (n-3) PUFAs in nonchallenged pigs and EPA in both challenged and nonchallenged pigs (*P* ≤ 0.001). MCT supplementation also led to a decrease in the (n-6)/(n-3) ratio in both challenged and nonchallenged pigs (*P* < 0.001).

### 2.3. MCT Supplementation Attenuates Liver Morphological and Ultrastructural Destruction Challenged by LPS

**Figure 1:**
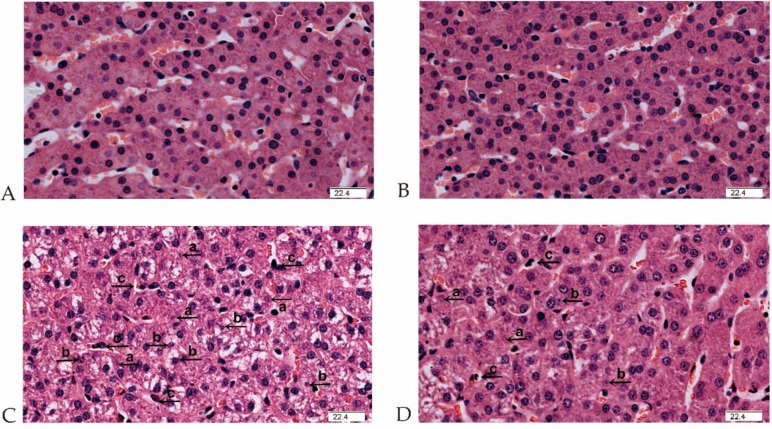
Effect of medium-chain triglyceride (MCT) supplementation on liver morphology after 4-h LPS challenge in weanling pigs. Pigs were subjected to a 2 × 2 factorial study fed with or without 4% MCTs for 21 days, followed by an intraperitoneal injection of saline or LPS on day 22. The representative liver histological sections from the four treatment groups were collected at 4 h after LPS challenge and stained with hematoxylin and eosin: (**A**) control diet and saline group, (**B**) 4% MCT diet and saline group, (**C**) control diet and LPS-challenged group and (**D**) 4% MCT diet and LPS-challenged group. While significant morphologic changes associated with liver injury, such as hepatocyte karyolysis (a), karyopyknosis (b), inflammatory cell infiltration (c) and disordered hepatic cell cords arrangement were observed in Panels **C** and **D**, significant attenuation of liver injury was observed in Panel **D**. Original magnification: 400×. Scale bars = 22.4 μm.

No obvious pathological changes were observed in nonchallenged pigs fed either the control diet (Figure 1A) or the MCTs (Figure 1B) by light microscopy examination. Obvious liver damage such as hepatocyte karyolysis, karyopyknosis, inflammatory cell infiltration and disordered hepatic cord arrangement were observed in LPS-challenged pigs fed the control diet (Figure 1C). Liver damage was clearly alleviated in LPS-challenged pigs fed the MCT-supplemented diet (Figure 1D).

**Figure 2:**
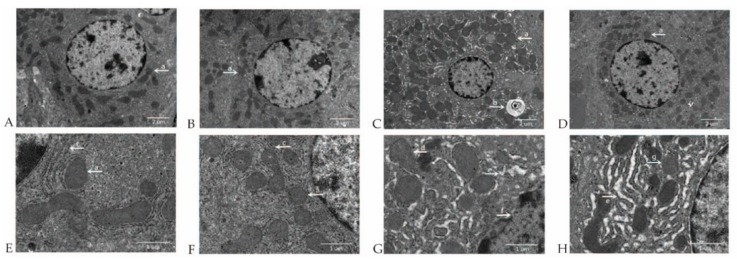
Effect of medium-chain triglyceride (MCT) supplementation on hepatocyte ultrastructure after 4-h LPS challenge in weanling pigs. Pigs were subjected to a 2 × 2 factorial study fed with or without 4% MCTs for 21 days, followed by an intraperitoneal injection of saline or LPS on day 22. The representative hepatocyte ultrastructural images from the four treatment groups were collected at 4 h after LPS challenge and processed for electron microscopy examination: (**A**) control diet and saline group, mitochondria (a), original magnification: 1700 ×, scale bars = 2 μm; (**B**) 4% MCT diet and saline group, mitochondria (a), original magnification: 1700 ×, scale bars = 2 μm; (**C**) control diet and LPS-challenged group, mitochondria (a), autophagosome (b), original magnification: 1700 ×, scale bars = 2 μm; (**D**) 4% MCT diet and LPS-challenged group, mitochondria (a), original magnification: 1700 ×, scale bars = 2 μm; (**E**) control diet and saline group, mitochondria (a), endoplasmic reticulum (c), original magnification: 5000 ×, scale bars = 1 μm; (**F**) 4% MCT diet and saline group, mitochondria (a), endoplasmic reticulum (c), original magnification: 5000 ×, scale bars = 1 μm; (**G**) control diet and LPS-challenged group, mitochondrial dissolution (d), endoplasmic reticulum expansion (e), nuclear deformation, nuclear membrane rupture and chromatin overflow (f), original magnification: 5000 ×, scale bars = 1 μm. (**H**) 4% MCT diet and LPS-challenged group, mild mitochondrial swelling (g), endoplasmic reticulum expansion (e) and integral nuclear membrane, original magnification: 5000 ×, scale bars = 1 μm.

Electron microscopy examination did not reveal significant pathologic changes within the hepatocytes of nonchallenged pigs. Normal hepatocyte morphology, clear cell boundary and integral nuclear membrane were observed in nonchallenged pigs fed either the control diet (Figure 2A,E) or MCT-supplemented diet (Figure 2B,F). Marked increase of hepatocyte mitochondria number, appearance of autophagosomes (Figure 2C), mitochondrial dissolution, endoplasmic reticulum expansion, nuclear deformation, nuclear membrane rupture and chromatin overflow (Figure 2G) were observed in LPS-challenged pigs fed the control diet. Hepatocytes ultrastructural damage was alleviated in LPS-challenged pigs fed the MCT-supplemented diet, which was proved by moderate increase of hepatocyte mitochondria number (Figure 2D), mild mitochondrial swelling and integral nuclear membrane (Figure 2H).

### 2.4. MCT Supplementation Decreases Serum Alanine Aminotransferase (ALT) and Alkaline Phosphatase (AKP) activities after LPS Challenge

No LPS challenge × diet interaction was observed for serum biochemical parameters (Table 1). At 2 h post injection, serum AKP (*P* = 0.09) and glutamyl transpeptidase (GGT) (*P* = 0.09) activities were higher in the LPS challenged group than in the saline group. Compared with the control diet, MCTs reduced serum ALT activity (*P* < 0.05) and tended to decrease AKP (*P* = 0.09) activity. At 4 h post injection, aspartate aminotransferase (AST), AKP and GGT activities were higher in pigs challenged by LPS (all *P ≤* 0.01) and the ALT/AST ratio was lower (*P* < 0.01) than pigs given saline. Relative to the pigs fed the control diet, pigs fed MCTs had lower serum ALT and AKP activities (*P* < 0.05).

### 2.5. MCT Supplementation Increases Liver Claudin-1 and Heat Shock Protein 70 (HSP70) Protein Expression after LPS Challenge

**Figure 3:**
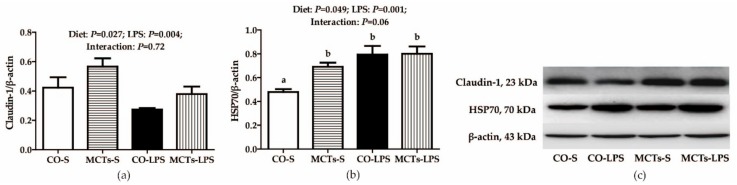
Effect of medium-chain triglyceride (MCT) supplementation on claudin-1 (**a**) and heat shock protein 70 (HSP70) (**b**) protein expression in the liver after 4-h LPS challenge in pigs. The bands are the representative Western blot images (**c**). Values are mean and SE, *n* = 6 (1 pig/pen). Means with different letters differ significantly (*P* < 0.05). All data for protein expression were acquired using Western blot. Values for relative claudin-1 and HSP70 expression were normalized for β-actin. CO-S, pigs fed the control diet and injected with saline; MCTs-S, pigs fed MCTs and injected with saline; CO-LPS, pigs fed the control diet and challenged with LPS; MCTs-LPS, pigs fed MCTs and challenged with LPS.

The LPS-challenged pigs had lower claudin-1 and higher HSP70 protein expression in the liver than saline-injected pigs (*P* < 0.01) (Figure 3). No LPS challenge × diet interaction was found for claudin-1. MCTs increased claudin-1 protein expression (*P* < 0.05). A trend for an LPS challenge × diet interaction was observed for HSP70 in the liver (*P* = 0.06). MCTs increased HSP70 protein expression in saline-injected pigs but had no effect in LPS-challenged pigs.

### 2.6. MCT Supplementation Decrease Serum and Liver Proinflammatory Cytokine Concentrations and Inhibits Liver TLR4, NODs and Their Downstream Signals after LPS Challenge

**Figure 4:**
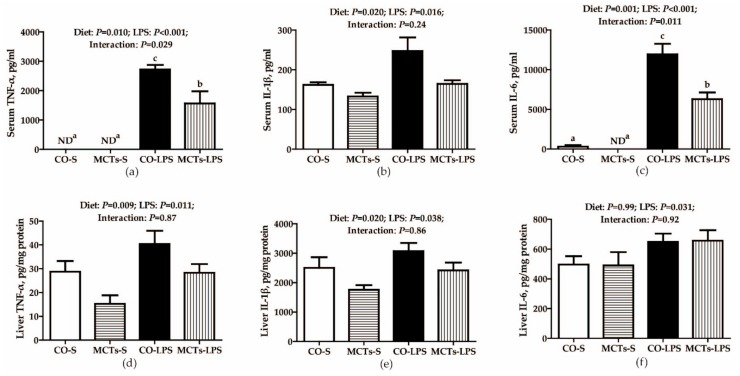
Effect of medium-chain triglyceride (MCT) supplementation on concentrations of serum (**a**–**c**) and liver (**d**–**f**) proinflammatory cytokines after 4-h LPS challenge in pigs. Values are mean and SE, n = 6 (1 pig/pen). Means with different letters differ significantly (*P* < 0.05). CO-S, pigs fed the control diet and injected with saline; MCTs-S, pigs fed MCTs and injected with saline; CO-LPS, pigs fed the control diet and challenged with LPS; MCTs-LPS, pigs fed MCTs and challenged with LPS. ND, not detectable, low than the minimum detectable doses of TNF-α and IL-6.

The LPS-challenged pigs had higher TNF-α, IL-1β and IL-6 concentrations in serum and liver (*P* < 0.05) (Figure 4). An LPS challenge × diet interaction was observed for serum TNF-α and IL-6 (*P* < 0.05). The concentrations of serum TNF-α and IL-6 in response to LPS challenge were lower in pigs fed MCTs than in those fed the control diet. No such effects were observed in pigs that received saline. No LPS challenge × diet interaction was found for serum IL-1β and hepatic TNF-α, IL-1β and IL-6. MCTs decreased the concentrations of serum IL-1β and hepatic TNF-α and IL-1β (*P* < 0.05).

Liver mRNA expression of myeloid differentiation factor (*MyD*) 88, *NOD2*, *RIP2*, *TNF-α*, *IL-1β* and *IL-6* (all *P* < 0.01) and *NOD1* (*P* = 0.06) in LPS-challenged pigs was higher compared with saline-injected pigs (Table 2). No LPS challenge × diet interaction was found for hepatic *MyD88*, TNF-α receptor-associated factor (*TRAF*) 6, *NOD1*, *NOD2*, *NF-κB*, or *IL-6* but MCTs reduced *MyD88*, *TRAF6*, *NOD1* and *NF-κB* mRNA expression (*P* < 0.05). An LPS challenge × diet interaction was observed for hepatic *RIP2*, *TNF-α* and *IL-1β* (*P* < 0.05) and a trend for an LPS challenge × diet interaction was observed for *TLR4* (*P* = 0.07) and IL-1 receptor-associated kinase (*IRAK*) 1 (*P* = 0.06). Hepatic mRNA expression of all these signaling molecules in response to LPS challenge was lower in pigs fed MCTs than in those fed the control diet. No such effects were observed in pigs that received saline.

**Figure 5:**
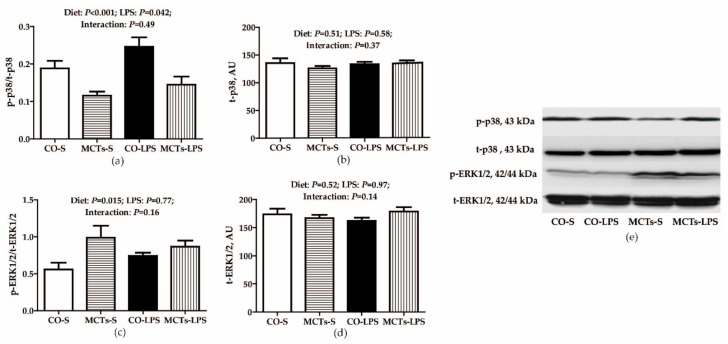
Effect of medium-chain triglyceride (MCT) supplementation on liver p38 (**a**,**b**) and extracellular signal-related kinase 1/2 (ERK1/2) (**c**,**d**) phosphorylation levels after 4-h LPS challenge in pigs. The bands are the representative Western blot images (**e**). Values are mean and SE, *n* = 6 (1 pig/pen). All data for protein expression were acquired using Western blot. Phosphorylated forms of p38 and ERK1/2 were normalized to the total amount of each protein. CO-S, pigs fed the control diet and injected with saline; MCTs-S, pigs fed MCTs and injected with saline; CO-LPS, pigs fed the control diet and challenged with LPS; MCTs-LPS, pigs fed MCTs and challenged with LPS. AU, arbitrary units.

No LPS challenge × diet interaction was observed for the phosphorylated p38/total p38 (p-p38/t-p38) and phosphorylated extracellular signal-related kinase 1/2/total extracellular signal-related kinase 1/2 (p-ERK1/2/t-ERK1/2) ratios (Figure 5). Compared with nonchallenged pigs, LPS-challenged pigs had a higher p-p38/t-p38 ratio in the liver (*P* < 0.05) but MCTs decreased the p-p38/t-p38 ratio (*P* < 0.001) and increased the p-ERK1/2/t-ERK1/2 ratio (*P* < 0.05) in liver compared with the control diet.

### 2.7. MCT Supplementation Inhibits Liver mRNA or Protein Expression of Necroptosis-signaling Molecules after LPS Challenge

Liver mRNA expression of *RIP1*, *MLKL* and phosphoglycerate mutase (*PGAM*) 5 (all *P* < 0.001) was higher and that of *RIP3* was lower (*P* < 0.05) in LPS-challenged pigs compared with saline-treated pigs (Table 3). No LPS challenge × diet interaction was observed for *RIP1* and *RIP3*, MCTs decreased *RIP3* mRNA expression relative to the control diet (*P* < 0.01). A diet × LPS interaction was observed for *MLKL* and Fas-associated death domain (*FADD*) (*P* < 0.01) and a trend for a diet × LPS interaction was observed for *PGAM5* (*P* = 0.09). LPS-challenged pigs fed the MCT diet had lower hepatic *MLKL* and *PGAM5* mRNA expression than pigs fed the control diet and saline-treated pigs fed the MCT diet had higher *FADD* mRNA expression compared with pigs fed the control diet.

**Figure 6:**
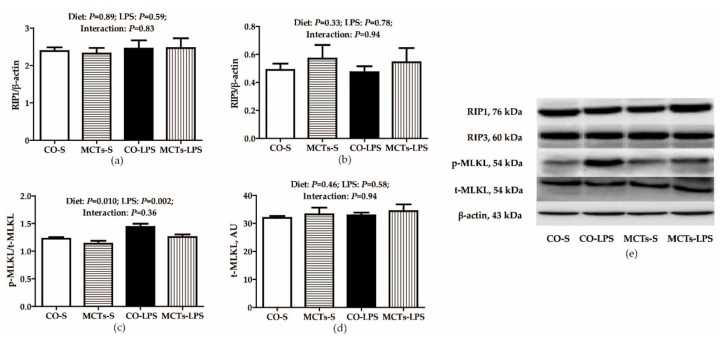
Effect of medium-chain triglyceride (MCT) supplementation on protein expression of key signaling molecules in necroptosis after 4-h LPS challenge in pigs (**a**–**d**). The bands are the representative Western blot images (**e**). Values are mean and SE, *n* = 6 (1 pig/pen). All data for protein expression were acquired using Western blot. Values for relative RIP1 and RIP3 expression were normalized against β-actin and the phosphorylated form of MLKL was normalized with total protein content of MLKL. CO-S, pigs fed the control diet and injected with saline; MCTs-S, pigs fed MCTs and injected with saline; CO-LPS, pigs fed the control diet and challenged with LPS; MCTs-LPS, pigs fed MCTs and challenged with LPS. RIP, receptor-interacting serine/threonine-protein kinase; MLKL, mixed-lineage kinase domain-like protein; AU, arbitrary units.

Neither diet nor LPS challenge influenced RIP1, RIP3, or total MLKL protein expression (Figure 6). No LPS challenge × diet interaction was observed for the phosphorylated MLKL/total MLKL (p-MLKL/t-MLKL) ratio. LPS significantly increased the p-MLKL/t-MLKL ratio (*P* < 0.01). Compared with the control diet, MCTs decreased the p-MLKL/t-MLKL ratio (*P* = 0.01).

## 3. Discussion

Because of shorter carbon chain lengths, medium-chain fatty acids have different pharmacokinetics and utilize different metabolic pathways compared with long-chain fatty acids. Medium-chain fatty acids are not incorporated into chylomicrons but are absorbed directly into the hepatic portal vein and rapidly metabolized for energy in both enterocytes and hepatocytes [8]. In addition to contributing to energy metabolism, MCTs have antimicrobial, anti-inflammatory, antioxidative and anti-obesity activities [23,24,25] and have hepatoprotective effects in alcoholic and nonalcoholic liver disease models [12,13]. This study investigated the hepatoprotective effects and molecular mechanisms of MCTs in a pig model of LPS-induced liver injury.

The hepatic fatty acyl synthase that catalyzes triglyceride re-esterification has a chain-length preference of 14 or more carbons. We observed no incorporation of C8–C10 fatty acids into liver lipids but replacement of corn oil with MCTs decreased linoleic acid and total (n-6) PUFA concentrations and the (n-6)/(n-3) ratio. It also increased EPA, DHA and total (n-3) PUFA concentrations, which is consistent with a previous report [13]. Because (n-3) PUFAs have anti-inflammatory activity [26], the MCT-mediated increase in hepatic (n-3) PUFAs in this study might have led to reduced liver susceptibility to inflammatory challenge.

Transaminases (AST and ALT) are intracellular hepatocyte enzymes and liver injury results in increases in serum AST and ALT activities [2,27]. Serum AKP and GGT activities are also markers of liver injury [2]. In this study, LPS challenge increased both serum AKP and GGT activities at 2 h post challenge and increased serum AST, AKP and GGT activities and reduced the ALT/AST ratio at 4 h. MCTs decreased serum ALT and AKP activities at both 2 and 4 h. These findings were consistent with the results of liver histological and ultrastructural examinations. LPS-challenged pigs had severe liver histological damage and hepatocyte ultrastructural alterations. Only mild liver histological damage and hepatocyte ultrastructural alterations were observed in MCT-fed pigs. Dietary MCT supplementation clearly alleviated the damage to hepatic morphology and function triggered by LPS, which is in line with another study finding MCTs inhibited increase of serum ALT and prevented pathologic changes in rat liver tissue after LPS administration [28]. Different from long-chain triglycerides, MCTs have specific nutritional and metabolic characteristics including rapid digestion, passive absorption and obligatory oxidation [8] and thus directly support hepatic integrity in weanling pigs.

Tight junctions are intercellular complexes that maintain tissue integrity and barrier function [29]. Disruption of tight junctions can lead to deterioration of barrier function and an increase in paracellular permeability [2]. Claudin-1 is a major integral membrane protein involved in maintaining tight junctions [30]. In this study, consistent with improved hepatic tissue architecture and function, MCTs increased the expression of claudin-1 protein. This result is similar to a previous finding that MCTs upregulated the expression of mRNAs coding for tight junction proteins (ZO-1, claudin-1 and occludin) and adaptor proteins (symplekin and fodrin) in the ileum associated with improved intestinal barrier integrity in a mouse model of alcoholic liver disease [31].

We also evaluated the impact of MCTs on the hepatic inflammatory response. Overproduction of TNF-α, IL-1β and IL-6 can lead to inflammation and liver injury [2,32]. HSPs are anti-inflammatory factors that mediate cytoprotective functions [33] and an increase in intracellular HSP70 can decrease the inflammatory response and promote liver regeneration [34]. In this study, MCTs were associated not only with improved liver structure and function but also with decreased serum TNF-α, IL-1β, IL-6 concentrations and decreased hepatic *TNF-α* and *IL-1β* mRNA expression and protein concentrations and increased HSP70 protein expression in the liver, clearly indicating the anti-inflammatory activity of MCTs. MCTs were previously found to decrease *TNF-α* mRNA expression in the liver, concurrent with reversal of alcohol-induced liver necrosis in a rat model [35].

TLR- and NOD-mediated activation of the NF-κB and MAPK signaling pathways triggers inflammation. We further observed that MCTs suppressed the LPS-induced mRNA expression of multiple molecules involved in TLR and NOD signaling, including *TLR4*, *NOD1*, *MyD88*, *IRAK1*, *TRAF6*, *RIP2* and *NF-κB*. In addition, dietary supplementation of MCTs decreased hepatic p38 MAPK phosphorylation. Therefore, the protective effect of MCTs on LPS-induced hepatic inflammation and damage can be partially attributed to the inhibition of TLR4 and NOD signaling and their downstream p38 MAPK pathway. MCTs were shown to down-regulate the expression of *TLR1*, *2*, *3*, *4*, *7*, *8* and *9* mRNAs in mouse liver after 8 weeks of alcohol feeding [31] and to inhibit the activation of hepatic NF-κB and p38 MAPK in high fat diet-induced obese mice [36]. In this study, MCT supplementation increased ERK1/2 phosphorylation, which conflicts with decreases of pro-inflammatory cytokines mRNA and protein expression. The reason for this discrepancy is not clear.

Necroptosis is a novel nonapoptotic form of programmed cell death that has been reported in many liver diseases [15]. Necroptosis can be triggered by extracellular stimuli such as TNF-α, Fas, TNF-related apoptosis-inducing ligand and TLR agonists that are known to trigger inflammation. Necroptosis is mediated by activation of RIP1 and RIP3, ultimately resulting in cell death and release of DAMPs [15,20,37], which further promote inflammation and secondary tissue injury [18]. Necroptosis inhibition has been shown to alleviate pathological changes in numerous animal models, providing a novel rationale for treatment of human diseases [38]. In this study, LPS challenge increased mRNA expression of *RIP1*, *MLKL* and *PAGM5* and protein phosphorylation of MLKL in the liver, consistent with a previous report by Nikseresht et al. [39]. Concurrent with less damage to hepatic integrity and decreased hepatic inflammation, MCT supplementation inhibited LPS-induced *RIP3*, *MLKL* and *PGAM5* mRNA expression and MLKL phosphorylation, indicating MCT inhibition of necroptosis.

Few studies have investigated nutritional modulation of necroptosis but DHA was found to reduce TNF-α-triggered necroptosis in L929 cells [40] by reducing oxidative stress, ceramide production, lysosomal dysfunction and autophagic features [41]. The available evidence suggests that (n-3) PUFAs such as DHA might inhibit necroptosis but animal studies are currently lacking. In this study, MCTs increased hepatic EPA, DHA and total (n-3) PUFA concentrations and decreased total (n-6) PUFA concentration and the (n-6)/(n-3) ratio. The increase in total (n-3) PUFA level in response to MCT supplementation partially explains how MCTs might suppress hepatic necroptosis.

In conclusion, dietary supplementation with MCTs alleviates hepatic damage induced by LPS in weanling pigs. The hepatoprotective effect of MCTs is associated with inhibition of necroptotic (RIP1/RIP3/MLKL) and inflammatory (TLR4/NOD1/p38 MAPK) signaling pathways.

## 4. Materials and Methods

### 4.1. Animal Care and Experimental Design

The research was conducted in accordance with the protocol approved by the Animal Care and Use Committee of Wuhan Polytechnic University (EM1226, 26 December 2016). Written informed consent was obtained from all participants. Twenty-four weanling pigs (Duroc × Large White × Landrace, 9.1 ± 0.2 kg) were randomly assigned to four treatment groups in a 2 × 2 factorial design. The pigs were individually housed in 1.80 × 1.10 m^2^ pens and allowed ad libitum access to feed and water. The ambient temperature was maintained at 22–25°C. The piglets were in good health condition and the living environment was in accordance with animal welfare guidelines in the whole experimental period. There were six replicate pens for each treatment. The pigs were fed a control diet (5% corn oil) or a MCT-supplemented diet (4% MCTs and 1% corn oil). MCTs and corn oil were provided by Lianyungang Huacheng Feed Company (Lianyungang, China) and Xiwang Food Company (Shandong, China), respectively. MCTs contained 53.02% caprylic acid (C8:0) and 46.20% capric acid (C10:0). Corn oil contained 12.41% palmitic acid (C16:0) and 29.72% oleic acid and 54.09% linoleic acid. The experimental diets (Table S2) met or exceeded NRC nutrient requirements for weanling pigs [42]. The fatty acid contents of the control and MCT-supplemented diets (Table S3) were measured by gas chromatography as previously described [43].

The experiment was arranged as a 2 × 2 factorial design including dietary treatment (0 and 4% MCTs) and immunological challenge (saline and LPS). After 3-weeks of feeding, the pigs in the challenge group were injected intraperitoneally with 100 μg/kg BW of LPS from *Escherichia coli* 055: B5 (#L2880, Sigma Chemical, St. Louis, MO, USA), while the remaining pigs were injected with the same volume of sterile saline on the morning of day 22. The LPS dose and the time of injection were chosen as previously described [2]. The pigs were weighed and feed intake was recorded on day 1 and 21.

### 4.2. Blood and Liver Sample Collection

Blood and liver samples were collected as previously described [2]. Briefly, blood samples were obtained at 2 and 4 h post injection. Serum was isolated by centrifugation (3500× g, 10 min) and then stored at −80°C until used. After blood collection at 4 h, the pigs were humanely killed by intravenous injection of pentobarbital sodium (80 mg/kg BW) for harvesting of liver tissue. One portion of each tissue sample was fixed in fresh 4% paraformaldehyde in phosphate-buffered saline for at least 24 h and then embedded in paraffin for histological examination. Another portion was cut into smaller pieces (about 1 mm^3^) and fixed with 2.5% glutaraldehyde at 4 °C for transmission electron microscopic examination. The remaining liver tissue was divided into smaller pieces and immediately frozen in liquid nitrogen and then stored at −80 °C for fatty acid profiling, mRNA and protein expression assays.

### 4.3. Assay of Liver Fatty Acid Composition

The fatty acid profiles of liver tissue were analyzed as described by Nieto et al. [43].

### 4.4. Liver Histological Examination

Serial 5 µm paraffin sections of liver tissue were deparaffinized and stained with hematoxylin and eosin for microscopic examination. Histological analysis was performed in a blinded manner by an experienced pathologist using a light microscope with a computer-assisted morphometric system (BioScan Optimetric, BioScan, Edmonds, WA, USA).

### 4.5. Ultrastructural Analysis of Hepatocytes

The liver tissues were dehydrated with alcohol, covered by epoxy resin, sliced into ultrathin layers, stained with uranyl acetate and lead citrate and examined with a transmission electron microscope (Tecnai G^2^ 20 TWIN, FEI, Eindhoven, the Netherlands) at an acceleration voltage of 200 kV.

### 4.6. Assay of Serum Biochemical Parameters

Serum AST, ALT, AKP and GGT activities were assayed as previously described [2].

### 4.7. Measurement of Serum and Liver Proinflammatory Cytokines

The concentrations of TNF-α, IL-6 and IL-1β in serum and liver supernatant were determined using commercially available porcine ELISA kits (TNF-α: #PTA00; IL-6: #P6000B; IL-1β: #PLB00B, R&D Systems, Minneapolis, MN, USA). The concentrations of TNF-α, IL-6 and IL-1β in liver supernatant were expressed as pg/mg protein.

### 4.8. Western Blot Assay of Protein Expression

Western blot assays were performed as previously described [2]. Briefly, 0.15–0.20 g liver samples were homogenized in lysis buffer and centrifuged to collect the supernatants. Hepatic proteins were then separated on polyacrylamide gels and transferred to polyvinylidene fluoride membranes. The membranes were blocked with 5% skim milk powder in tris-buffered saline/Tween-20 for at least 60 min at room temperature followed by sequential incubation with primary antibodies at 4°C overnight and secondary antibodies at room temperature for 120 min. Primary antibodies included rabbit anti-claudin-1 (1:1000; #51-9000, Invitrogen, Carlsbad, CA, USA), mouse anti-HSP70 (1:1000; #ADI-SPA-810, Enzo Life Sciences, Raamsdonksveer, the Netherlands), rabbit anti-RIP1 (1:1000; #LS-B8214, LifeSpan BioSciences, Seattle, WA, USA), rabbit anti-RIP3 (1:1000; #SC-135170, Santa Cruz Biotechnology, Santa Cruz, CA, USA), rabbit anti-p-MLKL (1:1000; #91689S, Cell Signaling Technology, Danvers, MA, USA), rabbit anti-t-MLKL (1:1000; #14993S, Cell Signaling Technology), rabbit anti-p-p38 (1:1000; #9211, Cell Signaling Technology), rabbit anti-t-p38 (1:1000; #9212, Cell Signaling Technology), rabbit anti-p-ERK1/2 (1:1000; #9101s, Cell Signaling Technology), rabbit anti-t-ERK1/2 (1:1000; #9102s, Cell Signaling Technology) and mouse anti-β-actin (1:10,000; #A2228, Sigma Aldrich, St. Louis, MO, USA). The secondary antibodies included goat anti-rabbit IgG-HRP (1:5000; #ANT020, Antgene Biotech, Wuhan, China) and goat anti-mouse IgG-HRP (1:5000; #ANT019, Antgene Biotech). Blots were developed, visualized and analyzed using an Enhanced Chemiluminescence Western Blotting Detection Kit (Amersham, Bucks, UK), Gene Genome Bioimaging System and GeneTools software (Syngene, Cambridge, UK), respectively. The relative expression of target proteins (claudin-1, HSP70, RIP1 and RIP3) was expressed as the target protein/β-actin ratio. Phosphorylated MLKL, p38 and ERK1/2 were normalized against the total amount of each protein.

### 4.9. Real-time PCR Assay of mRNA Expression

mRNA expression was assayed as previously described [2]. Isolation and quantification of total RNA (#9108, TRIzol reagent, TaKaRa Biotechnology, Dalian, China), cDNA synthesis (#RR047A, PrimeScript^®^ RT reagent kit, TaKaRa Biotechnology, Dalian, China) and real-time PCR (#RR420A, SYBR^®^ Premix Ex TaqTM (TliRNaseH Plus) qPCR kit, TaKaRa Biotechnology, Dalian, China) were conducted following the manufacturer’s instructions. The gene-specific primer sequences are shown in Table S4. mRNA expression of the target genes was normalized against a reference gene (*GAPDH*) using the 2^−ΔΔCT^ method [44].

### 4.10. Statistical Analysis

Data were analyzed by ANOVA using a general linear model appropriate for a 2 × 2 factorial design using SAS software (SAS Institute, Cary, NC, USA). The statistical model included the effects of immunological challenge (saline or LPS), dietary treatment (0 or 4% MCTs) and their interactions. If there was a significant interaction or an interaction trend, post hoc testing was conducted using Duncan’s multiple comparison. *P* ≤ 0.05 was considered as statistically significant; 0.05 < *P* ≤ 0.10 was considered as to be a trend.

## Figures and Tables

**Table 1:** Effect of medium-chain triglyceride (MCT) supplementation on serum biochemical parameters after 2- or 4-h LPS challenge in pigs *.

Item	Saline		LPS		SEM	*P* Value		
	Control	MCTs	Control	MCTs		Diet	LPS	Interaction
**2 h**								
ALT, U/L	73.2	60.1	73.1	50.1	8.0	0.035	0.53	0.54
AST, U/L	97	92	97	88	15	0.64	0.88	0.90
ALT/AST	0.783	0.815	0.775	0.633	0.128	0.67	0.47	0.50
AKP, U/L	279	233	319	279	24	0.09	0.09	0.90
GGT, U/L	37.3	44.0	45.2	57.0	6.1	0.14	0.09	0.69
**4 h**								
ALT, U/L	75.7	62.4	71.7	52.7	7.0	0.033	0.34	0.69
AST, U/L	85	92	159	160	25	0.88	0.010	0.91
ALT/AST	0.895	0.850	0.543	0.435	0.131	0.57	0.009	0.81
AKP, U/L	280	242	422	304	29	0.013	0.002	0.18
GGT, U/L	34.5	43.2	63.2	74.0	7.3	0.20	0.001	0.89

* Values are mean and pooled SEM, *n* = 6 (1 pig/pen). ALT, alanine aminotransferase; AST, aspartate aminotransferase; AKP, alkaline phosphatase; GGT, glutamyl transpeptidase.

**Table 2:** Effect of medium-chain triglyceride (MCT) supplementation on mRNA expression of inflammation-related signaling molecules after 4-h LPS challenge in pigs *^,†^.

Item	Saline		LPS		SEM	*P* Value		
	Control	MCTs	Control	MCTs		Diet	LPS	Interaction
*TLR4*	1.00 ^ab^	0.92 ^a^	1.70 ^b^	0.71 ^a^	0.24	0.035	0.31	0.07
*MyD88*	1.00	0.84	1.93	1.16	0.18	0.020	0.003	0.11
*IRAK1*	1.00 ^ab^	0.87 ^a^	1.47 ^b^	0.70 ^a^	0.16	0.012	0.37	0.06
*TRAF6*	1.00	0.80	1.15	0.69	0.14	0.032	0.90	0.38
*NOD1*	1.00	0.77	1.78	0.84	0.21	0.013	0.06	0.11
*NOD2*	1.00	1.41	7.03	6.19	0.60	0.72	<0.001	0.31
*RIP2*	1.00 ^a^	0.77 ^a^	7.55^c^	3.87 ^b^	0.70	0.011	<0.001	0.023
*NF-κB*	1.00	0.77	1.40	0.88	0.17	0.045	0.16	0.42
*TNF-α*	1.00 ^a^	1.43 ^ab^	4.25 ^c^	2.28 ^b^	0.35	0.040	<0.001	0.003
*IL-1β*	1.00 ^a^	1.04 ^a^	47.38 ^c^	20.36 ^b^	5.74	0.029	<0.001	0.029
*IL-6*	1.00	1.16	14.13	14.20	2.41	0.96	<0.001	0.98

* Values are mean and pooled SEM, *n* = 6 (1 pig/pen). Labeled means in a row without a common letter differ, *P* < 0.05. TLR4, toll-like receptor 4; MyD88, myeloid differentiation factor 88; IRAK1, IL-1 receptor-associated kinase 1; TRAF6, TNF-α receptor-associated factor 6; NOD, nucleotide-binding oligomerization domain protein; RIP2, receptor-interacting serine/threonine-protein kinase 2; NF-κB, nuclear factor-κB. ^†^ All the data were acquired using real-time PCR normalized against GAPDH as the housekeeping gene.

**Table 3:** Effect of medium-chain triglyceride (MCT) supplementation on mRNA expression of key signaling molecules in necroptosis after 4-h LPS challenge in pigs *^,†^.

Item	Saline		LPS		SEM	*P* Value		
	Control	MCTs	Control	MCTs		Diet	LPS	Interaction
*RIP1*	1.00	0.79	2.77	2.74	0.24	0.62	< 0.001	0.72
*RIP3*	1.00	0.72	0.81	0.38	0.12	0.007	0.040	0.56
*MLKL*	1.00 ^a^	0.94 ^a^	12.23 ^c^	5.36 ^b^	1.17	0.008	< 0.001	0.009
*FADD*	1.00 ^a^	1.63 ^b^	1.54 ^ab^	1.12 ^ab^	0.18	0.57	0.93	0.009
*PGAM5*	1.00 ^a^	0.89 ^a^	2.79 ^c^	1.82 ^b^	0.24	0.037	<0.001	0.09

* Values are mean and pooled SEM, *n* = 6 (1 pig/pen). Labeled means in a row without a common letter differ, *P* < 0.05. RIP, receptor-interacting serine/threonine-protein kinase; MLKL, mixed-lineage kinase domain-like protein; FADD, Fas-associated death domain; PGAM5, phosphoglycerate mutase 5. ^†^ All the data were acquired using real-time PCR normalized against GAPDH as the housekeeping gene.

## Supplementary Materials

Supplementary materials can be found at http://www.mdpi.com/1422-0067/19/11/3697/s1.
