# Supplementary material for: Medium-Chain Triglycerides Attenuate Liver Injury in Lipopolysaccharide-Challenged Pigs by Inhibiting Necroptotic and Inflammatory Signaling Pathways
Source: Int J Mol Sci. 2018 Nov 21;19(11):3697. doi: 10.3390/ijms19113697 (PMC6274951; doi:10.3390/ijms19113697)
Supplement: Supplementary file 1 [file ijms-19-03697-s001.pdf]

Supplementary Materials

Table S1. Effect of medium-chain triglyceride (MCT) supplementation on liver fatty acid composition after 4-h LPS challenge in pigs *,†.

Item	Saline		LPS		SEM	*P* value		
	Control	MCTs	Control	MCTs		Diet	LPS	Interaction
			% total fatty acids					
C6:0	0.51	0.57	0.47	0.51	0.03	0.17	0.11	0.69
C16:0	25.86	25.67	26.08	25.91	0.28	0.52	0.43	0.97
C16:1	1.07	1.89	1.11	1.74	0.08	<0.001	0.50	0.30
C18:0	15.36	15.41	15.68	15.63	0.17	0.99	0.15	0.76
cis C18:1n-9	17.79 ^{ab}	18.85^{bc}	17.06 ^{a}	19.81 ^{c}	0.40	<0.001	0.77	0.06
cis C18:2n-6	17.42	14.10	16.45	13.49	0.71	0.001	0.29	0.81
C18:3n-3	0.25	0.28	0.22	0.25	0.02	0.18	0.18	0.88
C20:4n-6	10.29	10.60	11.14	10.19	0.56	0.58	0.70	0.28
C20:5n-3	0.24 ^{a}	0.52^{c}	0.31 ^{a}	0.41 ^{b}	0.02	<0.001	0.29	0.002
C22:6n-3	1.60 ^{a}	2.07^{c}	1.77 ^{ab}	1.81 ^{b}	0.06	0.001	0.42	0.003
Total (n-6) PUFAs‡	28.99	26.01	28.94	24.98	0.40	<0.001	0.21	0.25
Total (n-3) PUFAs‡	2.24 ^{a}	3.03^{c}	2.44 ^{ab}	2.60 ^{b}	0.08	<0.001	0.18	0.002
(n-6)/(n-3)‡	13.00 ^{d}	8.59^{a}	11.29 ^{c}	9.61 ^{b}	0.27	<0.001	0.24	<0.001

* Values are mean and pooled SEM, n = 6 (1 pig/pen). Labeled means in a row without a common letter differ, $P < 0.05$. † The liver fatty acid profiles from C4:0 to C24:1n-9 were analyzed in duplicate. Only the major fatty acids are listed. ‡ Total (n-6) or (n-3) PUFAs corresponded to the sum of all (n-6) or (n-3) PUFAs detected.

Table S2. Ingredient composition of diets (%, as-fed basis).

Ingredients		Nutrient level †	
Corn	56.00	Digestible energy (MJ/Kg)	14.0
Soyabeans	22.00	Crude protein	20.2
Wheat bran	3.00	Ca	0.90
Fish meal	5.50	Total P	0.70
Corn oil or Medium-chain triglycerides	5.00	Lysine	1.35
Soybean protein concentrate	2.50	Methionine + Cystine	0.72
Whey powder	3.00		
Limestone	0.70		
Dicalcium phosphate	1.00		
Salt	0.20		
L-Lysine HCl	0.27		
Acidifier	0.20		
Antioxidant	0.05		
Preservative	0.05		
Sweeteners	0.03		
Vitamin and mineral premix *	0.50		

* Premix supplied per kg diet: retinyl acetate, 5512 IU; cholecalciferol, 2200 IU; DL-α-tocopheryl acetate, 30 IU; menadione sodium bisulfite complex, 4 mg; riboflavin, 5.22 mg; D-calcium-pantothenate, 20 mg; niacin, 26 mg; vitamin B_{12}, 0.01 mg; Mn ($MnSO_4 \cdot H_2O$), 40 mg; Fe ($FeSO_4 \cdot H_2O$), 75 mg; Zn ($ZnSO_4 \cdot 7H_2O$), 75 mg; Cu ($CuSO_4 \cdot 5H_2O$), 100 mg; I (CaI_2), 0.3 mg; Se (Na_2SeO_3), 0.3 mg. † The nutrients level was analyzed value except digestible energy which is calculated value.

Table S3. Fatty acid profiles of the control and medium-chain triglyceride (MCT)-supplemented diets *,†.

Fatty acid	Control diet	MCT-supplemented diet
	% total fatty acids	
C6:0	0.01	0.09
C8:0	0.04	20.22
C10:0	0.02	17.82
C16:0	14.32	9.84
C18:0	2.38	1.82
cis C18:1n-9	27.34	15.47
cis C18:2n-6	50.61	29.78
C18:3n-3	1.39	1.16
C20:4n-6	0.11	0.12
C20:5n-3	0.37	0.40
C22:6n-3	0.86	0.99
Total (n-6) PUFAs ‡	50.72	29.90
Total (n-3) PUFAs ‡	1.71	2.55
(n-6)/(n-3) ‡	29.66	11.73

* Control diet: 5% corn oil; MCT-supplemented diet: 4% MCTs and 1% corn oil. † The liver fatty acid profiles from C4:0 to C24:1n-9 were analyzed in duplicate. Only the major fatty acids are listed. ‡ Total (n-6) or total (n-3) PUFAs are the sum of all detected (n-3) or (n-6) PUFAs.

Table S4. Gene-specific primer sequences used in real-time PCR *.

Gene	Primer sequence (5'-3')	PCR product (bp)	Accession numbers
TLR4	F: TCAGTTCTCACCTTCCTCCTG<br>R: GTTCATTCCTCACCCAGTCTTC	166	GQ503242.1
MyD88	F: GATGGTAGCGGTTGTCTCTGAT<br>R: GATGCTGGGGAACTCTTTCTTC	148	AB292176.1
IRAK1	F: CAAGGCAGGTCAGGTTTCGT<br>R: TTCGTGGGGCGTGTAGTGT	115	XM_003135490.1
TRAF6	F: CAAGAGAATACCCAGTCGCACA<br>R: ATCCGAGACAAAGGGGAAGAA	122	NM_001105286.1
NOD1	F: CTGTCGTCAACACCGATCCA<br>R: CCAGTTGGTGACGCAGCTT	57	AB187219.1
NOD2	F: GAGCGCATCCTCTTAACTTTCG<br>R: ACGCTCGTGATCCGTGAAC	66	AB195466.1
RIP2	F: CAGTGTCCAGTAAATCGCAGTTG<br>R: CAGGCTTCCGTCATCTGGTT	206	XM_003355027.1
NF-κB	F:AGTACCCTGAGGCTATAACTCGC<br>R: TCCGCAATGGAGGAGAAGTC	133	EU399817.1
TNF-α	F: TCCAATGGCAGAGTGGGTATG<br>R: AGCTGGTTGTCTTTCAGCTTCAC	67	NM_214022.1
IL-1β	F:GCTAACTACGGTGACAACAATAATG<br>R:CTTCTCCACTGCCACGATGA	186	NM_214055.1
IL-6	F: AAGGTGATGCCACCTCAGAC<br>R:TCTGCCAGTACCTCCTTGCT	151	JQ839263.1
RIP1	F: ACATCCTGTACGGCAACTCT<br>R: CGGGTCCAGGTGTTTATCC	175	XM_005665538.2
RIP3	F: CTTGTTGTCTGTCCGTGAGC<br>R: GAGGAGGTTGGGCTGTTGA	238	XM_001927424.3
MLKL	F: TCTCGCTGCTGCTTCA<br>R: CTCGCTTGTCTTCCTCTG	105	XM_013998184.1
FADD	F: AAGTGTCTGACGCCAAG<br>R: CCTCCTGCTGTTCTTCC	101	XM_013987237.1
PGAM5	F: TCTTCATCTGCCACGCCAAT<br>R: GGTGATGCTGCCGTTGTTG	104	XM_013992365.1
GAPDH	F: CGTCCCTGAGACACGATGGT<br>R: GCCTTGACTGTGCCGTGGAAT	194	AF017079.1

* F, forward; R, reverse. TLR4, toll-like receptor 4; MyD88, myeloid differentiation factor 88; IRAK1, IL-1 receptor-associated kinase 1; TRAF6, TNF-α receptor-associated factor 6; NOD, nucleotide-binding oligomerization domain protein; RIP, receptor-interacting serine/threonine-protein kinase; NF-κB, nuclear factor-κB; MLKL, mixed-lineage kinase domain-like protein; FADD, Fas-associated death domain; PGAM5, phosphoglycerate mutase 5.
